# The neurophysiology of the intervention strategies of Awareness Training Program on emotion regulation

**DOI:** 10.3389/fpsyg.2022.891656

**Published:** 2022-07-22

**Authors:** Junling Gao, Hang Kin Leung, Jicong Fan, Bonnie Wai Yan Wu, Hin Hung Sik

**Affiliations:** ^1^Buddhism and Science Research Lab, Centre of Buddhist Studies, The University of Hong Kong, Pokfulam, Hong Kong SAR, China; ^2^School of Data Science, The Chinese University of Hong Kong, Shenzhen, China

**Keywords:** emotion regulation, event-related potential, Awareness Training Program, compassion and wisdom meditation, stress, heart rate variability

## Abstract

Emotion regulation is essential for healthy living. Previous studies have found that mental training such as compassion meditation could help with emotion regulation. However, the underlying neural mechanism and possible intervention strategies of group-based Mahayana Buddhist intervention involved in emotion regulation are still unclear. This event-related potential (ERP) study investigated how compassion and wisdom meditations, two key components of the Awareness Training Program (ATP), may regulate emotion during different mental processing stages, namely attention deployment, cognitive change, and response modification. Eighty-five middle-aged working adults with moderate stress were voluntarily recruited for this study, using a 128-channel electroencephalogram system. After 7 weeks of training, participants (ATP attendance, *n* = 42; waitlist control, *n* = 43) were instructed to view negative pictures while practicing compassion or wisdom meditation, with corresponding priming words. Another normal priming condition and a neutral picture condition were set as control conditions. ERP results in the ATP group showed that negative pictures induced greater prefrontal activity (N400 component) in both compassion and wisdom meditation conditions compared with the normal condition, while the control group showed little difference between the conditions. Significantly higher heart rate variability was found in the compassion but not wisdom meditation when compared with the neutral priming condition. Correspondent changes in behavioural data were also found. Converging evidence showed that compassion meditation training could modulate negative emotion processing in stages of attention deployment, cognitive change, and behavioural responses. The prefrontal lobe could play an important role in the process of emotion regulation by compassion meditation, possibly due to the emphasis of the ATP on contemplative practices.

## Introduction

Emotion regulation is an adaptive function essential for a healthy working and living. For evolutionary purposes, the human brain is more responsive to negative events than to neutral or pleasant events. However, this tendency could lead to negative bias, delusive rumination, and even affective disorders (Rozin and Royzman, 2001; Hilgard et al., 2014). Given the stressful environment associated with contemporary life, it is necessary to explore practical methods of emotion regulation and, more importantly, to understand the working mechanism and how these methods modulate emotion in different stages of affective information processing.

Affective information is sequentially processed by different brain regions. Correspondingly, emotion can be regulated in one or more stages to modulate/intercept affective information processing flow, resulting in various intervention strategies. According to the Gross process model, one can modulate emotion at different stages. Externally, the process of situation selection and modification can be employed. Internally, after having engaged with the situation, the individual can regulate their emotions *via* attention deployment, cognitive change, and modulation of response expression (Gross, 2002; Goldenberg et al., 2016).

Mindfulness training has become popular and has broad applications. Numerous studies have demonstrated that mindfulness meditation helps with emotion regulation in various populations, including patients with post-traumatic stress disorder, attention deficit hyperactivity disorder (ADHD), depression, or psychopathy (Freudenthaler et al., 2017; Mitchell et al., 2017; Tang, 2018; Reffi et al., 2019). A mindfulness-based stress reduction (MBSR) programme emphasizes being non-judgmental and stopping to pay attention to the ongoing emotional information (Catak and Ogel, 2010; Murakami et al., 2015). Thus, it may regulate the emotion in the stage of attention deployment, according to Gross’s process model of emotion regulation (Gross, 2002; Goldenberg et al., 2016). Nevertheless, other intervention strategies can also be quite effective in emotion regulation. For example, cognitive reappraisal can be used in the cognitive change stage (Zilverstand et al., 2017; Dryman and Heimberg, 2018). Previous research has shown that cognitive change may be more effective for emotion regulation than other strategies like distraction and suppression (Thiruchselvam et al., 2011; Grezellschak et al., 2015). In addition, research has revealed that promoting awareness, understanding, and acceptance are adaptive strategies of emotion regulation, while avoidance and control of emotion are maladaptive strategies (Aldao, 2013; Tull et al., 2020).

Mindfulness meditation is currently the most popular means of mental training. Nevertheless, other mental training exists in meditation traditions and may also be effective in terms of helping people to regulate and cope with emotional disturbance and stress (Lopez and Pellegrini, 2011; Desbordes et al., 2015; Gao et al., 2017; Bayot et al., 2020). From a Buddhist point of view, meditation generally can promote concentration, mental clarity and awareness and, subsequently, tranquilize emotional turbulence. The practice of meditation has two parts: tranquillity (*śamatha*) and observation (*vipaśyanā*). When practiced in-depth concurrently, these two can lead to the attainment of ultimate enlightenment (John, 2000).

We thus designed the Awareness Training Program (ATP) to enhance negative emotion regulation by incorporating compassion and wisdom training. It is assumed that the development of compassion and wisdom of non-attachment as taught by Mahayana Buddhism could induce positive cognitive changes through better understanding and reappraisal of negative events. The ATP incorporated three pedagogical steps: learning, contemplation, and practice of developing wisdom as taught in the *Sandhinirmochana Sūtra*, the classic and most important text on meditation methods in the *Yogācāra* school of Mahayana Buddhism (John, 2000). The ATP is a group-based intervention that could efficiently enhance participants’ emotion regulation and stress management. More details on the theoretical foundation, the experimental design, and the practice of the ATP can be found in a previous behavioural study (Wu et al., 2019).

In the current study, we examined the neural mechanism of emotion regulation induced by the ATP, especially its two key components: compassion and wisdom training. Usually, the affective process involves a responsive process induced by external or internal stimuli in a bottom-up manner (Anderson et al., 2003). In contrast, the ATP intervention may modulate emotional information processing in a top-down manner (Challis and Berton, 2015). Bottom-up generation of emotion is more related to amygdala and adjacent limbic structures, while top-down regulations come more from dorsolateral PFC and anterior cingulate cortex (ACC). The brain regions for bottom-up and top-down processing are dissociable (Stenson et al., 2021), while the connectivity between these neocortical and limbic areas increases during emotion regulation. Additionally, ventral medial PFC can release 5-HT in the forebrain and enhance dorsal raphe nucleus to release 5-HT and subsequently restore emotion balance (Veerakumar et al., 2014; Challis and Berton, 2015). The compassion and wisdom meditations taught in the ATP required cognitive contemplation to enhance emotion regulation, therefore we assumed that the prefrontal cortex (PFC) may play an essential role in the mental contemplation during the ATP practices.

A majority of neuroimaging studies have investigated the neural mechanism of mindfulness in stress reduction. A functional magnetic resonance imaging (fMRI) study has reported that when participants anticipated depressing pictures, a mindfulness intervention could increase prefrontal activity and downregulate the activity of the amygdala, which is involved in emotional processing (Lutz et al., 2014). Another event-related potential (ERP) study has indicated that dispositional mindfulness can attenuate the neural response to both highly unpleasant and erotic images (Brown et al., 2013). Other studies have also suggested that mindfulness helps to modulate emotion-related brain networks (Allen et al., 2012). A previous electroencephalographic (EEG) study on mindfulness training has revealed that this approach is effective in decreasing the irregularity of electrical brain activity, as indicated by entropy, possibly because of the sense of calm and peacefulness that is produced during mindfulness breathing (Gao et al., 2016).

Few studies, however, have explored the neural mechanism of compassion meditation training and its mechanism on emotion regulation (Kim et al., 2009; Engstrom and Soderfeldt, 2010). Compassion meditation engages specific patterns of neural activation, including the anterior cingulate cortex, insula, and PFC (Lutz et al., 2008, 2009). The results of an fMRI meta-analysis of compassion did not demonstrate consistent results, probably due to a small number of studies on compassion, empathy or loving-kindness meditation (Kim et al., 2020). There is also a lack of understanding on the strategies used for emotion regulation. The literature review likewise did not yield any neuroscientific research on the wisdom of non-attachment meditation. Given the excellent temporal resolution of ERPs, it could be used to investigate emotion regulation in different stages and the related temporal dynamics (Thiruchselvam et al., 2011; Koval et al., 2015). A previous ERP study on emotion regulation has found that manipulation of attention deployment takes place earlier than manipulation of cognitive change (Thiruchselvam et al., 2011). The location of the ERP component can also provide spatial information regarding the brain areas involved in various stages of emotion regulation. Furthermore, through the application of head models and the conversion of surface signals, ERP source analysis can further localize the related electrical activity in the brain (Heidlmayr et al., 2015; Gao et al., 2017). In a previous ERP study on religious chanting, brain responses to frightening images, in terms of late-positive potential, largely disappeared while chanting the name of Amitabha Buddha, a prevalent spiritual practice in East Asia (Gao et al., 2017). However, the change only happened in the late stage but not in the early stage of negative emotion information processing.

We hypothesized that the ATP intervention could improve an individual’s emotion regulation ability, and the PFC would be involved in these modulations of affective information processing (Ochsner et al., 2004), probably during the cognitive change stage of emotion regulation. As a result of these modulations, we also hypothesized that as compared to the control group, the ATP group would show an improved resilience to stress and corresponding physiological and behavioural changes.

## Materials and methods

### Design of the intervention and data collection

In the current ERP study, 85 participants (ATP, *n* = 42; waitlist control, *n* = 43) were recruited from the previous randomized clinical trial (RCT) on the ATP; that is, these participants took part in the previous RCT study on the ATP and the current ERP study on the ATP (Wu et al., 2019). The mean age was 45.00 ± 8.00 and 46.67 ± 7.80 years old for the ATP and control groups, respectively. The two groups had no difference in mean age, gender, education level, marital status, and religious beliefs (see Supplementary Table S2). The experiment flow can be found in Supplementary Figure S1.

EEG data were collected from the ATP group after they completed the 7-week ATP course, and the data from the waitlist control group were obtained at a similar point in time. The experiment was conducted in a quiet room at an EEG laboratory. The EEG data were recorded using a 128-channel EGI™ machine (Electrical Geodesics, Inc. United States), with the impedance set below 30 kΩ. A LabChart™ system (PowerLab, ADInstrument Inc., Australia) was used to record other physiological data, including the ECG, pulse, and galvanic skin response (GSR), following the standard procedure.^1^ The ECG electrodes were placed using a limb lead. Pulse was measured at the left thumb, and GSR was measured on the left middle and index fingers.

The ERP experiment was designed to examine the effect of compassion/wisdom meditation on negative emotion regulation, respectively. It had four conditions: (1) a compassion condition (COMP, watching negative pictures with priming of a compassion cue); (2) a wisdom condition (WISD, watching negative pictures with priming of a wisdom cue); (3) a neutral condition (NEUT, watching negative pictures with priming of a neutral cue); and (4) a normal control condition (NORM, watching neutral pictures with priming of a neutral cue). This design followed the design of a previous emotion regulation study (Thiruchselvam et al., 2011), which had three regulating conditions for negative pictures and one additional normal condition as a baseline. Because emotions usually arise and last for a relatively long time (Hilgard et al., 2014), a block design was used. Each condition had one block and lasted for approximately 4 min. The sequence of the four conditions was randomized for different participants to avoid sequential effects.

**Figure 1:**
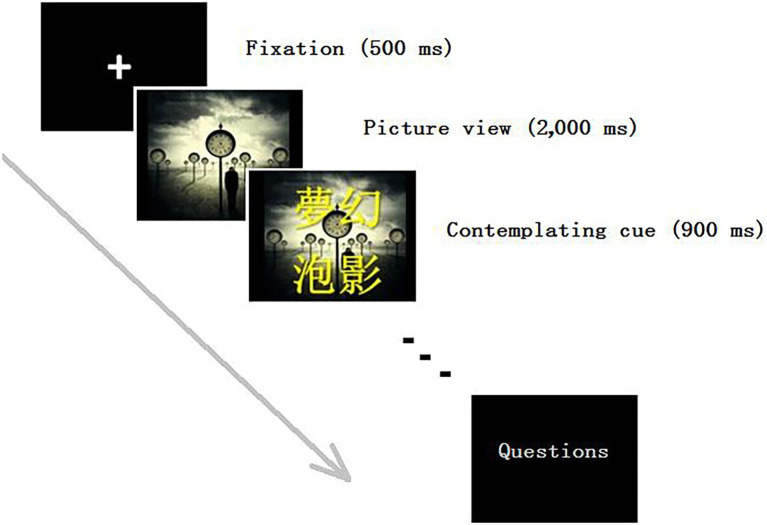
Design and timeline in the wisdom-compassion event-related potential experiment.

E-prime (Psychology Software Tools, Inc., United States) was used to display the pictures in the affective regulation task. The pictures were partly selected from the International Affective Picture System (IAPS), with additional valence-matched pictures from the Internet included. The negative pictures showed sad and miserable scenes of people, so as to easily induce negative emotion. At the beginning of the process for each condition, a brief passage regarding compassion, wisdom, or neutral instruction was presented for participants to contemplate. Thereafter, the participants were first shown a fixation (+), followed by a miserable picture that was displayed for 2,000 ms, followed by a phrase related to the earlier teaching to serve as a priming reminder. The inter-stimulus interval (3,400 ms) was chosen based on a previous paper on emotion regulation (Gao et al., 2017). Sixty trials of the same type of pictures were displayed consecutively in each condition, followed by three questions on how the participants felt (see details below). These questions needed to be answered within 20 s (the average answer time was about 7 s). Each participant went through all four conditions in random order (see Figure 1). The entire ERP experiment lasted approximately 30 min.

The behavioural, EEG, and physiological data were analysed offline with SPSS, MATLAB, EEGLAB, heart rate variability (HRV) analysis software (HRVAS), and statistical parametric mapping (SPM). The data from the three types of measurements were analysed separately.

### ERP data analysis

The ERP data were analysed by using EEGLAB, a toolbox based on MATLAB. The data were first pre-processed before final statistical comparisons were made to test our hypothesis. For pre-processing, EEG data were first resampled from 1,000 to 250 Hz; then, they were filtered by using a 30-Hz low-pass filter. EEG artefacts were removed by visually inspecting and deleting the EEG segment and channel data affected by apparently poor scalp-surface contact or excessive noise. Bad channels were reconstructed by using spherical interpolation. The independent component analysis method first generated the component data (IC), and around 10 to 20 ICs of eye blinks, bad channel, and muscle artefacts were discarded. Then, the event-related epochs were extracted and visually checked again for data quality. Finally, ERP data were generated by averaging the same types of epochs. The ERP map was re-referenced to a virtual reference with averaging of the potentials recorded at the left and right mastoids before statistical analysis.

The ERPs of the pictures were processed for each condition. The effect of compassion meditation and wisdom meditation was obtained by deducting the ERP of the neutral condition from the ERP of the compassion condition and wisdom meditation condition, respectively. These ERP data were then entered in the second-level analysis on group difference. ERP components within specific time windows were chosen for different stages of emotion regulation. P1/N170 and P2 components were assumed as early information processing in the attention deployment stage (Meaux et al., 2014), and N400 was identified as the later cognitive stage of emotion regulation, together with other time windows and components according to the ERP response and the literature (Herzmann et al., 2010; Kutas and Federmeier, 2011; Onitsuka et al., 2013; Nie and Yu, 2021). Independent *t*-tests were carried out to compare the differences between the groups in the compassion meditation condition and then in wisdom meditation condition, respectively.

### ERP source analysis

Source analysis was implemented with a toolbox named SPM12,^2^ based on the MATLAB platform (MathWorks Inc., United States). The procedures were as follows: (1) EEGLAB data were converted to the SPM12-readable format; (2) the EEG sensor positions were linked to the coordinate system of MRI (MNI coordinates), which is a process known as *co-registration*; (3) using the Greedy search-based algorithm, an inverse reconstruction of the scalp electrode signals into the three-dimensional (3D) brain source signals was carried out; and (4) a factorial design was used to investigate the main effects and the group × condition interaction. Please refer to our previous work for more details (Gao et al., 2017).

### Physiological data analysis

Data analysis of ECG, breath intervals, and GSR was performed for each participant and each condition using LABchart and MATLAB. Raw ECG data were cleaned *via* a Butterworth bandpass filter. The inter-beat interval was extracted following the replacement of outliers – three standard deviations away from the mean – *via* spline interpolation. Using the HRVAS toolbox,^3^ we detrended the inter-beat interval data and computed the time domain features of the HRV. The derived HRV metrics, specifically the root mean square of the successive differences (RMSSD), were subjected to statistical testing using SPSS 24.0. Finally, mixed ANOVA and *post hoc* tests were utilized to assess differences between groups, conditions, and their interaction. The alpha level of significance was set at 0.05.

### Behavioural data analysis

Demographic features (age, gender) of the participants and subjective behavioural responses after the picture viewing at the end of each block were analysed *via* analysis of variance (ANOVA) with SPSS. The subjective behavioural responses measured how the participants felt after viewing the pictures from the current block *via* three consecutive questions: (1) How stressed do you feel? (2) How miserable or sad do you think the people in the pictures feel? (3) To what degree do you feel you want to help the persons in the pictures? The responses were based on a 7-point Likert scale and recorded by E-prime software.

## Results

### Results of ERP picture viewing

**Figure 2:**
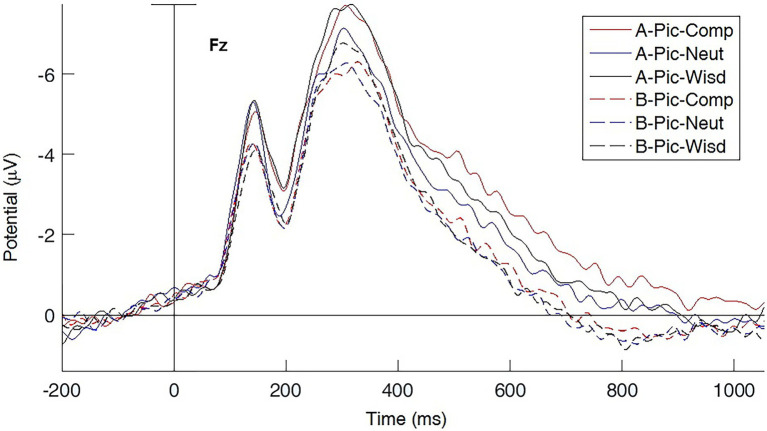
The event-related potentials of different priming conditions for negative pictures (Fz channel). The solid lines represent the ATP group and the dashed line is for the control group. ATP, Awareness Training Program; COMP, compassion meditation instruction; NEUT, neutral instruction; WISD, wisdom meditation instruction. A-, ATP group; B-, control group.

The ERP results illustrated the temporal processing of negative pictures during different intervenal conditions, and further second-level analysis was performed between groups. Among the specified ERP components, the early P1/N170 components are involved in information processing in the perceptual stage, while N170 and P2 are correlated with an individual’s emotional skills (Meaux et al., 2014). Therefore, the early components could be regarded as the attention deployment stage. The later stage contains more cognitive and affective processing of information in N400–600, and the component beyond 600 ms could be regarded as the cognitive change stage of emotion regulation. As the cognitive contemplation training of the ATP could influence frontal activity, we selected one channel in the frontal lobe (Fz) to observe and analyse the effects of the conditions in two groups when viewing the negative pictures (see Figure 2).

**Figure 3:**
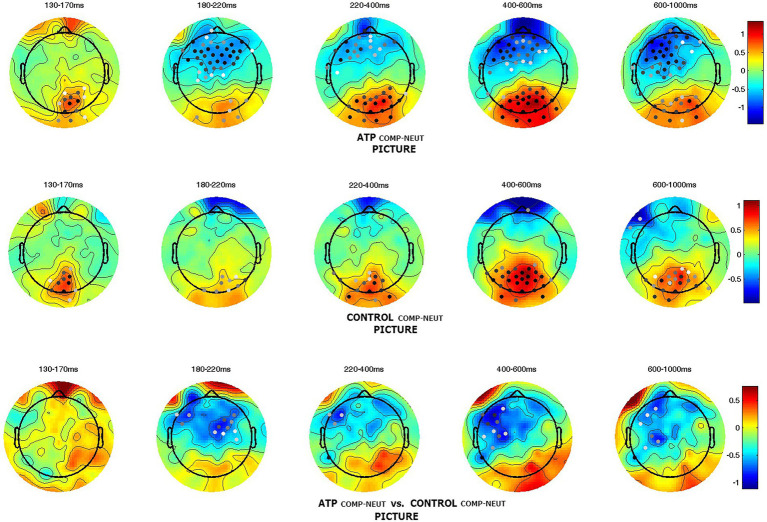
Group comparison of components in the compassion condition when viewing negative pictures. The patterns demonstrate that the Awareness Training Program (ATP) group had higher amplitudes (N400) in the left frontocentral region than the control group. Dots illustrate channels with significant differences (*p*    < 0.05), and darker dots indicate smaller *p*-values.

To further illustrate the effect of compassion and wisdom meditations, brain activity in these two meditation conditions was compared with that for the neutral priming condition. The effect of compassion meditation on a participant’s responses to miserable pictures was calculated by subtracting the neutral instruction condition from the compassion meditation condition (compassion – neutral); second-level comparisons between groups are shown in the bottom row of Figure 3. The outcome demonstrated that compassion meditation elicited higher brain activity at the earlier stage of negative picture processing, and this activity lasted for a longer time in the ATP group than in the control group. When they viewed miserable pictures with compassion priming (COMP), the ATP group had higher brain activity in the frontal and occipital lobes (see Supplementary Figures S2a,b), whereas the control group only had higher occipital activity (see Supplementary Figures S3a,b). To further compare the overall effect of COMP, region of interest (ROI) analysis was performed for the N400 component of channels in the left frontal regions (Control: *M* = −0.082 μV, *SD* = 1.606 μV; ATP: *M* = −0.822 μV, *SD* = 1.739 μV; *t* = 2.039, *p* = 0.045; ROI of the left frontal region, see Supplementary Figure S4).

**Figure 4:**
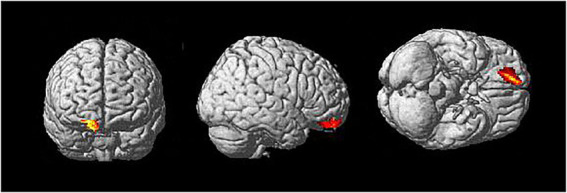
Source analysis on the comparison of compassion meditation effect between the Awareness Training Program (ATP) group and control group. Source analysis using statistical parametric mapping (SPM) indicated that the brain regions specifically activated by compassion meditation are located in the medial prefrontal region.

To further localize the brain region related to the compassion meditation (compassion-neutral condition) on miserable pictures, a source analysis was applied to illustrate the difference between the ATP and control groups. Source analysis suggested that the brain region involved in the compassion meditation condition is located in the medial prefrontal region (Figure 4).

**Figure 5:**
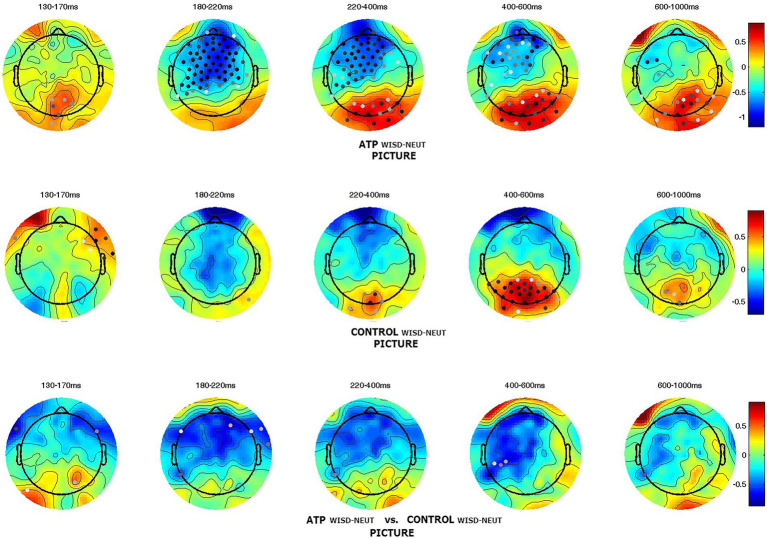
Group differences in the wisdom condition when viewing negative pictures. The Awareness Training Program (ATP) group had more neural activity (around N200–600 ms) in the frontal region than the control group. The dots illustrate channels with significant differences (*p* < 0.05), and darker dots indicate smaller *p*-values.

Wisdom meditation had a similar effect as compassion meditation on the frontal lobe, although the significance was less marked. In addition, this frontal activity occurred slightly earlier than in the compassion condition (see Supplementary Figures S5a,b). At the same time, the control had only more occipital activity when compared to the neutral condition (see Supplementary Figures S6a,b). The pattern in Figure 5 illustrates that, with the wisdom meditation priming, the ATP group had more frontal activity than the control group during negative picture viewing.

### Physiological results

**Figure 6:**
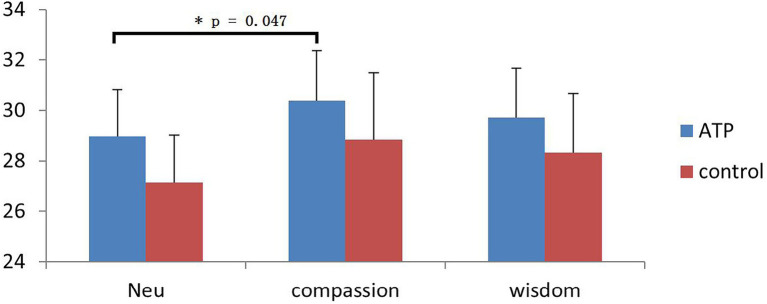
Group comparison of heartrate variability (HRV) under different conditions. The HRV is higher in the compassion condition than in the neutral condition in the Awareness Training Program (ATP) group. The error bar shows the standard error.

The ECG results indicated that when viewing negative pictures, the ATP group had a higher HRV in terms of the RMSSD, *t*(40) = −2.05, *p =* 0.047 (see Figure 6), in the COMP condition (*M* = 30.38) than in the NEUT condition (*M* = 28.97). Nonetheless, no significant difference in the heart rate interval during picture viewing between groups or conditions, and no significant effects were found for the GSR and breathe data (see Supplementary Table S1).

### Behavioural results

There was no difference in age or gender between the two groups (age of ATP group = 45.0 ± 8.0 years old; age of control group = 46.7 ± 7.8 years old). There was an apparent difference between the negative pictures (for the COMP, WISD, and NEUT conditions) and neutral pictures (NORM condition) when both groups answered the three subjective questions (*p* < 0.001). The significance of these results validated the separation between neutral and negative categories of pictures.

Further comparisons of subjective responses to the negative pictures indicated that the participants in the ATP group perceived the negative pictures as more miserable than the control group. However, the difference was significant only in the COMP condition (*p* = 0.042) and not in the WISD (*p* = 0.754) and NEUT (*p* = 0.947) conditions. The ATP group was less stressed than the control group, although the results were not statistically significant.

**Figure 7:**
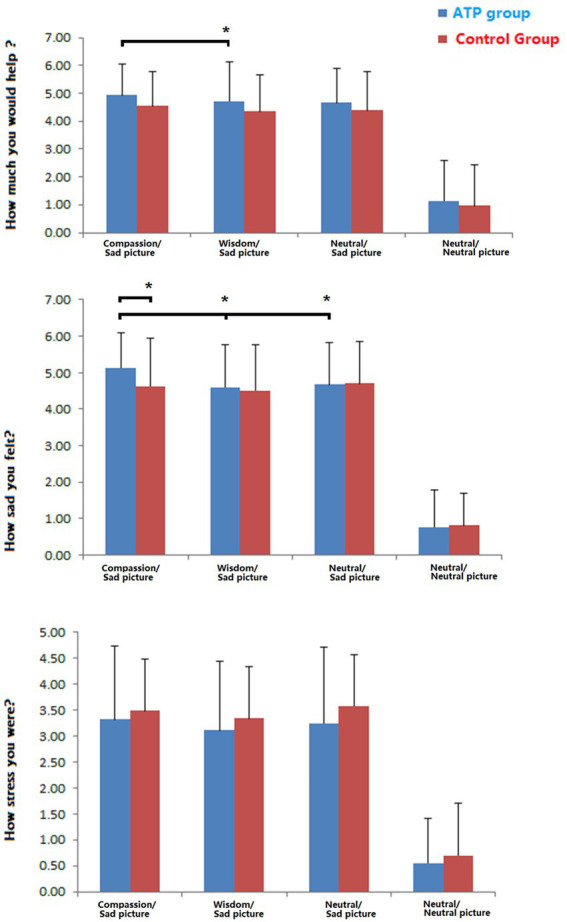
Behavioural measurements and comparison of how the participants felt when viewing the negative pictures. Note that all the scores are significantly different between negative and neutral pictures (*p*  < 0.001). ATP, Awareness Training Program. significant difference (*p  < 0.05).

The participants perceived negative pictures as significantly more miserable in the COMP condition than in the WISD (p < 0.001) and NEUT (*p* = 0.003) conditions, but only in the ATP group. In addition, the results demonstrated that the ATP group was more willing to help, but only in the COMP condition, compared with the WISD condition (*p* = 0.009). Similarly, the ATP group tended to be less stressed in the WISD condition than in the COMP condition (*p =* 0.094). In contrast, these differences between conditions were not significant in the control group, as shown in Figure 7.

## Discussion

Several behavioural studies, including ours, have shown the effectiveness of compassion or loving-kindness meditation on emotion regulation under challenging situations (Blevins, 2016; Newham, 2017; Inwood and Ferrari, 2018; Wu et al., 2019). However, these studies lack clarity with regard to the emotion regulation strategies used and the underlying neural mechanism. The current ERP study demonstrated the neural correlates of emotion regulation by using different priming after the 7-week practice of awareness training. Based on the neurophysiological results, we discuss how the ATP intervention, especially compassion meditation, affects the three aspects of negative emotion processing: attention deployment, cognitive change, and response expression.

### Effect of compassion/wisdom meditation on attention deployment

Attention deployment involves the early processing of perceptual information in the occipital lobe and the subsequent attentional neural activity in the frontal and parietal lobes (P1/N170 components). Compared with the NEUT condition, the ATP group showed greater P1/N170 component activity when viewing miserable pictures in both the COMP and WISD conditions. The control group did not show these early-stage neural activities in the COMP or WISD condition. Nevertheless, for these compassion-specific neural activities, the difference between the ATP group and the control group was not significant.

With primes of compassion/wisdom meditation, significantly more occipital activity was found in both the COMP and WISD conditions compared with the NEUT condition. This happened in the control and ATP groups. This finding indicates that participants in both groups tried to pay more attention when asked to show compassion or wisdom while viewing the negative and miserable pictures. Although participants of the control group were not explicitly taught how to meditate with compassion or wisdom, they did make efforts to contemplate following the primes of compassion or wisdom meditation. Thus, the control group also demonstrated more occipital activity in the COMP and WISD conditions. The ATP group showed slightly greater occipital activity than the control group, but the difference between the groups was not significant. This results is in line with previous findings that visual areas such as occipital lobe is mainly stimulus driven as shown in retinotopy, while parietal and frontal areas are driven primarily by attention. Nonetheless, the attentional modulation does affect both early visual areas and attentional control areas in parietal and frontal cortex (Saygin and Sereno, 2008).

The ATP course teaches participants to cultivate awareness and attention. Furthermore, compassion training is obligatory in the ATP, as the mind of enlightenment (*bodhicitta*) has compassion as its foundation. This may help to explain that after the compassion training, participants tended to be more prepared and to display an earlier and stronger willingness to respond in the COMP condition. That said, the insignificant results revealed that there was a trend that the awareness of the ATP participants might enhance after the training on compassion and wisdom meditation. However, the difference between groups was not evident at the early stage of attention deployment. The effect of compassion mediation/wisdom meditation may be well beyond the attention deployment stage.

### Effect of compassion/wisdom meditation on cognitive change

The cognitive change stage involved a period of later information processing at N400, and it could go beyond 600 ms to 1,000 ms, mainly in the frontal lobe. The most prominent finding in the ATP group was a significant amount of frontal activity after the priming of compassion meditation or wisdom meditation. In contrast, no such frontal activity was found in the control group. This outcome revealed that cognitive contemplation and the corresponding cognitive change were cultivated after practices of compassion and wisdom, and it played an important role in enhancing the ATP participants’ ability of affective modulation.

The frontal negativity of the ATP group peaked at 400–600 ms, a period generally referred to as an N400 component. The period of this cognitive process could last from 200 to 1,000 ms in some situations (Curran et al., 1993; Kutas and Federmeier, 2011; Joyal et al., 2020; Junge et al., 2021). The N400 component is highly relevant to language and semantic processing. Nonetheless, a review paper has suggested that N400 indicates fundamental processing of meaningful/non-meaningful dimension more than the linguistic/non-linguistic dimension (Kutas and Federmeier, 2011). The change in N400 amplitude in the COMP and WISD conditions demonstrates that both the compassion and wisdom practices could help individuals to reconceptualize the meaning of events but in different ways.

During compassion meditation or loving-kindness meditation, practitioners need to send blessings to themselves, and to others, especially those in trouble (Salzberg, 1995; Ho and Mak, 2016; Condon and Makransky, 2020; Mantzios et al., 2021). This contemplation during the compassion training could prompt more linguistic processing and help the participants to reconceptualize the experience and meaning of the miserable pictures. The result demonstrated that the ATP group had a higher N400 amplitude in the COMP condition than the control group. With primes of compassion meditation, this greater activation of the left frontal lobe in the COMP condition might imply a motivation to help and possibly an approach tendency (Davidson et al., 2004; Harmon-Jones et al., 2006), as the left frontal activity is related to an approach tendency (Kelley et al., 2017). The results are consistent with a previous ERP study on compassion, which revealed the role of frontal lobe in compassion-focussed reappraisal (Baker et al., 2017).

Further source analysis revealed that in the COMP condition, the ATP group demonstrated high neural activity in the medial PFC. There is evidence that the PFC can modify emotion according to socially acceptable norms by top-down processes (Morawetz et al., 2016). Damage to the frontal lobe or the pathways connecting the frontal lobe with the limbic system can disrupt the subject’s emotion, volition, and personality (Riva et al., 2012). Our results are concordant with previous studies demonstrating that compassion meditation might induce activity in the medial PFC and anterior cingulate cortex in response to empathy and happy feelings (Engstrom and Soderfeldt, 2010). A meta-analysis of fMRI studies on compassion also revealed compassion related areas at frontal lobe including the anterior cingulate, the inferior frontal gyrus, anterior insular, as well as other brain regions of periaqueductal grey, left putamen, left thalamus (Kim et al., 2020). Interestingly, this study found more regions were induced by compassion in the left hemisphere than the right hemisphere. The practice of compassion meditation cultivates the feeling of compassion and generosity, and practitioners need to cultivate their relationships with others despite whether or not they originally liked it. This cognitive reappraisal process may engage the middle and ventral areas of the PFC (Gusnard et al., 2001).

A similar frontal N400 component was found in the WISD condition, although it peaked earlier (200–400 ms) and lasted from 200 to 600 ms. In contrast, frontal negativity peaked at 400–600 ms and lasted from 200 to 1,000 ms in the COMP condition. These ERP results revealed that the frontal activity induced by miserable pictures in the WISD condition emerged earlier but lasted for less time compared with that in the COMP condition. This may reflect different contemplation between the COMP and WISD conditions in the stage of cognitive change. Cognitive contemplation was applied in both the WISD and COMP conditions, but their contemplative focus and content may differ. The wisdom meditation taught in the ATP aims to cultivate non-attachment, a Buddhist concept based on the empty nature of the ‘self’ or ‘other’ (Shiah, 2016). This insight may help individuals to let go of mental fixation or attachment to specific concepts. Thus, the participants could avoid excessive emotional responses and demonstrate an earlier peak and a shorter duration of the frontal activity in the WISD condition. It is suggested that wisdom practice cultivates a detach tendency instead of an avoid tendency. While in the COMP condition, the ATP participants may learn to cultivate an approach tendency. They tended to be cognitively more considerate, as implied by their prolonged prefrontal activity in the COMP condition.

### Effect of compassion/wisdom meditation on the ERP during the response modulation stage

The response modulation stage of participants could be observed in their behavioural response and physiological response of cardiac activity. The behavioural response showed that the ATP group displayed more empathy and willingness to help those in miserable situations than did the control group. However, this willingness to help seems only apparent in the COMP condition but not in other conditions. It is interesting to find that only in the ATP group, participants became more empathic to those in miserable situations in the COMP condition compared with the other two conditions, also they were more willing to help in the COMP condition than in the WISD condition. This finding shows that the priming cue could help the participants to retrieve their learning experience from the ATP training and enabled them to specifically reconceptualize the emotional pictures.

The ATP group had a higher HRV (in terms of the RMSSD) in the COMP condition than in the NEUT condition. As cardiac activity is modulated by the autonomic nervous system, which controls one’s physiological response to stress, HRV reflects the balance between the sympathetic nervous system and the parasympathetic nervous system (Singh et al., 2018). For example, HRV has a significant correlation with highly negative emotions induced by the IAPS pictures (Choi et al., 2017). A higher HRV in the compassion condition implies a higher degree of emotional involvement when viewing the miserable pictures. Changes in the physiological data suggest the cultivation of empathy in the compassion meditation condition.

A previous study showed that cognitive reappraisal could induce cardiac-vagal flexibility and cultivate positive emotion. Nonetheless, this effect only appeared after habitual use – that is, a relatively long-term training. Otherwise, the effect on HRV would be less prominent, similarly to the effect of expressive suppression (Jentsch and Wolf, 2020). The physiological change may be accompanied by a possible change in neuroendocrine activity, as self-reported compassion towards sad faces induces greater activation in subcortical areas secreting dopamine, including the ventral tegmental area and substantia nigra, generating an intrinsic reward (Kim et al., 2009). Previous studies have demonstrated that the PFC could influence neuroendocrine activity when regulating extreme emotions *via* the cognitive reappraisal strategy (Sullivan, 2004; Zhan et al., 2017). This empathetic understanding of other’s pain and the willingness to help may be related to the increased activation of the medial PFC (Immordino-Yang et al., 2009), as illustrated in our source analysis.

It is worth noting that to exercise fully the effectiveness of the cognitive reappraisal strategy of the ATP, both compassion and wisdom should be equally emphasized. Compassion training alone may entail an issue of compassion fatigue or secondary traumatic stress (van Mol et al., 2015). The simultaneous practice with the wisdom of nonattachement would allow the individual to step outside of their usual immediate response to an event and disentangle the habitual response of emotion from behaviour (Ayduk and Kross, 2010). In Buddhist teachings, compassion and wisdom meditations are inseparable, similarly to two wings of a bird. The current ERP study implies that compassion practice might endow a miserable event with more meaning and importance of life, thus cultivating an approach tendency. At the same time, wisdom practice might enable the participants to become acentric from a secular event and thus cultivate a detach tendency in the stage of cognitive reappraisal.

In sum, the current ERP study has demonstrated that both compassion meditation and wisdom meditation help affective modulation in middle-aged working adults with moderate stress. The behavioural results, physiological results, and ERP, especially the distinct frontal activity, provide converging evidence that the ATP could effectively regulate individuals’ emotional responses to miserable events. These results align with previous studies that cognitive reappraisal is an especially promising strategy of emotion regulation. That said, it has been suggested that the combination of compassion and wisdom may maximise the efficacy of ATP intervention in emotion regulation, and the combination could help sustain cognitive transformation towards compassion together with the wisdom of non-attachment (Salzberg, 2011).

Several limitations are worth noting in the current study. Traditional awareness training usually takes much longer than 7 weeks. Longer programme duration may result in a more effective outcome, especially for wisdom meditation, which is more difficult to learn and practice. Second, subcortical regions such as the amygdala play a vital role in emotion generation, and subsequent regulation, and neuroimaging tools such as fMRI could clarify this processing better than EEG source localisation in terms of spatial resolution. A third limitation is that we did not collect the ERP data before the ATP course, a two-arm longitudinal randomized clinical trial would be more convincing. The present study has demonstrated the role of frontal activity in compassion meditation. Future fMRI studies could reveal the subcortical regions involved in the bottom-up process of emotion regulation.

## Data availability statement

The original contributions presented in the study are included in the article/Supplementary Material, further inquiries can be directed to the corresponding authors.

## Ethics statement

The studies involving human participants were reviewed and approved by Human Research Ethics Committee The University of Hong Kong. The patients/participants provided their written informed consent to participate in this study.

## Author contributions

JG designed and executed the study, analysed and interpreted the data, and wrote the manuscript. HL analysed and interpreted the ERP and physiological data, and collaborated in manuscript writing. JF collected and analysed the ERP data. BW developed the ATP intervention, recruited the participants, assisted in the study design, interpretation of the data and manuscript writing. HS developed the ATP intervention, collaborated on the direction of the study, and assisted in the study design, interpretation of the data and manuscript writing. All authors contributed to the article and approved the submitted version.

## Funding

This research was funded by the Li Ka Shing Foundation.

## Conflict of interest

The authors declare that the research was conducted in the absence of any commercial or financial relationships that could be construed as a potential conflict of interest.

## Publisher’s note

All claims expressed in this article are solely those of the authors and do not necessarily represent those of their affiliated organizations, or those of the publisher, the editors and the reviewers. Any product that may be evaluated in this article, or claim that may be made by its manufacturer, is not guaranteed or endorsed by the publisher.

## Supplementary material

The Supplementary Material for this article can be found online at:

https://www.frontiersin.org/articles/10.3389/fpsyg.2022.891656/full#supplementary-material

## Footnotes

^1^ www.adinstruments.com/support/manuals

^2^ http://www.fil.ion.ucl.ac.uk/spm/software/spm12/

^3^ https://sourceforge.net/projects/hrvas

## References

[ref1] Aldao A. (2013). The future of emotion regulation research: capturing context. Perspect. Psychol. Sci. 8, 155–172. doi: 10.1177/1745691612459518 26172497

[ref2] Allen M. Dietz M. Blair K. S. van Beek M. Rees G. Vestergaard-Poulsen P.. (2012). Cognitive-affective neural plasticity following active-controlled mindfulness intervention. J. Neurosci. 32, 15601–15610. doi: 10.1523/Jneurosci.2957-12.2012, PMID: 23115195 PMC4569704

[ref3] Anderson A. K. Christoff K. Panitz D. De Rosa E. Gabrieli J. D. (2003). Neural correlates of the automatic processing of threat facial signals. J. Neurosci. 23, 5627–5633. doi: 10.1523/JNEUROSCI.23-13-05627.2003, PMID: 12843265 PMC6741280

[ref4] Ayduk O. Kross E. (2010). From a distance: implications of spontaneous self-distancing for adaptive self-reflection. J. Pers. Soc. Psychol. 98, 809–829. doi: 10.1037/a0019205, PMID: 20438226 PMC2881638

[ref5] Baker J. C. Williams J. K. Witvliet C. V. O. Hill P. C. (2017). Positive reappraisals after an offense: event-related potentials and emotional effects of benefit-finding and compassion. J. Posit. Psychol. 12, 373–384. doi: 10.1080/17439760.2016.1209540

[ref6] Bayot M. Vermeulen N. Kever A. Mikolajczak M. (2020). Mindfulness and empathy: differential effects of explicit and implicit Buddhist teachings. Mindfulness 11, 5–17. doi: 10.1007/s12671-018-0966-4

[ref7] Blevins C. L. (2016). Mindful compassion: how the science of compassion can help you understand your emotions, live in the present, and connect deeply with others. Mindfulness 7, 1246–1248. doi: 10.1007/s12671-016-0553-5

[ref8] Brown K. W. Goodman R. J. Inzlicht M. (2013). Dispositional mindfulness and the attenuation of neural responses to emotional stimuli. Soc. Cogn. Affect. Neurosci. 8, 93–99. doi: 10.1093/scan/nss004, PMID: 22253259 PMC3541486

[ref9] Catak P. D. Ogel K. (2010). Mindfulness as a therapy method. Noropsikiyatri Ars. 47, 69–73.

[ref10] Challis C. Berton O. (2015). Top-Down control of serotonin systems by the prefrontal cortex: a path toward restored Socioemotional function in depression. ACS Chem. Neurosci. 6, 1040–1054. doi: 10.1021/acschemneuro.5b00007, PMID: 25706226 PMC4504761

[ref11] Choi K. H. Kim J. Kwon O. S. Kim M. J. Ryu Y. H. Park J. E. (2017). Is heart rate variability (HRV) an adequate tool for evaluating human emotions? A focus on the use of the international affective picture system (IAPS). Psychiatry Res. 251, 192–196. doi: 10.1016/j.psychres.2017.02.025, PMID: 28213189

[ref12] Condon P. Makransky J. (2020). Sustainable compassion training: integrating meditation theory with psychological science. Front. Psychol. 11:2249. doi: 10.3389/fpsyg.2020.02249, PMID: 33041897 PMC7518715

[ref13] Curran T. Tucker D. M. Kutas M. Posner M. I. (1993). Topography of the N400 – brain electrical-activity reflecting semantic expectancy. Electroencephalogr. Clin. Neurophysiol. 88, 188–209. doi: 10.1016/0168-5597(93)90004-9, PMID: 7684968

[ref14] Davidson R. J. Shackman A. J. Maxwell J. S. (2004). Asymmetries in face and brain related to emotion. Trends Cogn. Sci. 8, 389–391. doi: 10.1016/j.tics.2004.07.006, PMID: 15350238

[ref15] Desbordes G. Gard T. Hoge E. A. Holzel B. Kerr C. Lazar S. W.. (2015). Moving Beyond mindfulness: defining equanimity as an outcome measure in meditation and contemplative research. Mindfulness 6, 356–372. doi: 10.1007/s12671-013-0269-8, PMID: 25750687 PMC4350240

[ref16] Dryman M. T. Heimberg R. G. (2018). Emotion regulation in social anxiety and depression: a systematic review of expressive suppression and cognitive reappraisal. Clin. Psychol. Rev. 65, 17–42. doi: 10.1016/j.cpr.2018.07.004, PMID: 30064053

[ref17] Engstrom M. Soderfeldt B. (2010). Brain activation during compassion meditation: a case study. J. Altern. Complement. Med. 16, 597–599. doi: 10.1089/acm.2009.0309, PMID: 20804370

[ref18] Freudenthaler L. Turba J. D. Tran U. S. (2017). Emotion regulation mediates the associations of mindfulness on symptoms of depression and anxiety in the general population. Mindfulness 8, 1339–1344. doi: 10.1007/s12671-017-0709-y, PMID: 28989550 PMC5605587

[ref19] Gao J. L. Fan J. C. Wu B. W. Halkias G. T. Chau M. Fung P. C.. (2017). Repetitive religious chanting modulates the late-stage brain response to fear and stress-provoking pictures. Front. Psychol. 7:2055. doi: 10.3389/fpsyg.2016.02055, PMID: 28119651 PMC5223166

[ref20] Gao J. Fan J. Wu B. W. Zhang Z. Chang C. Hung Y. S.. (2016). Entrainment of chaotic activities in brain and heart during MBSR mindfulness training. Neurosci. Lett. 616, 218–223. doi: 10.1016/j.neulet.2016.01.001, PMID: 26784361

[ref21] Goldenberg A. Halperin E. van Zomeren M. Gross J. J. (2016). The process model of group-based emotion: integrating intergroup emotion and emotion regulation perspectives. Personal. Soc. Psychol. Rev. 20, 118–141. doi: 10.1177/1088868315581263, PMID: 25870386

[ref22] Grezellschak S. Lincoln T. M. Westermann S. (2015). Cognitive emotion regulation in patients with schizophrenia: evidence for effective reappraisal and distraction. Psychiatry Res. 229, 434–439. doi: 10.1016/j.psychres.2015.05.103, PMID: 26231583

[ref23] Gross J. J. (2002). Emotion regulation: affective, cognitive, and social consequences. Psychophysiology 39, 281–291. doi: 10.1017/S0048577201393198, PMID: 12212647

[ref24] Gusnard D. A. Akbudak E. Shulman G. L. Raichle M. E. (2001). Medial prefrontal cortex and self-referential mental activity: relation to a default mode of brain function. Proc. Natl. Acad. Sci. U. S. A. 98, 4259–4264. doi: 10.1073/pnas.071043098, PMID: 11259662 PMC31213

[ref25] Harmon-Jones E. Lueck L. Fearn M. Harmon-Jones C. (2006). The effect of personal relevance and approach-related action expectation on relative left frontal cortical activity. Psychol. Sci. 17, 434–440. doi: 10.1111/j.1467-9280.2006.01724.x, PMID: 16683932

[ref26] Heidlmayr K. Hemforth B. Moutier S. Isel F. (2015). Neurodynamics of executive control processes in bilinguals: evidence from ERP and source reconstruction analyses. Front. Psychol. 6:821. doi: 10.3389/fpsyg.2015.00821, PMID: 26124740 PMC4467069

[ref27] Herzmann G. Kunina O. Sommer W. Wilhelm O. (2010). Individual differences in face cognition: brain-behavior relationships. J. Cogn. Neurosci. 22, 571–589. doi: 10.1162/jocn.2009.21249, PMID: 19400675

[ref28] Hilgard J. Weinberg A. Proudfit G. H. Bartholow B. D. (2014). The negativity bias in affective picture processing depends on top-down and bottom-up motivational significance. Emotion 14, 940–949. doi: 10.1037/a0036791, PMID: 24866528 PMC4172529

[ref29] Ho H. H. Mak W. W. S. (2016). Effects of cognitive understanding and practice of loving-kindness meditation on well-being. Int. J. Psychol. 51, 672.

[ref30] Immordino-Yang M. H. McColl A. Damasio H. Damasio A. (2009). Neural correlates of admiration and compassion. Proc. Natl. Acad. Sci. U. S. A. 106, 8021–8026. doi: 10.1073/pnas.0810363106, PMID: 19414310 PMC2670880

[ref31] Inwood E. Ferrari M. (2018). Mechanisms of change in the relationship between self-compassion, emotion regulation, and mental health: a systematic review. Appl. Psychol. Health Well Being 10, 215–235. doi: 10.1111/aphw.12127, PMID: 29673093

[ref32] Jentsch V. L. Wolf O. T. (2020). The impact of emotion regulation on cardiovascular, neuroendocrine and psychological stress responses. Biol. Psychol. 154:107893. doi: 10.1016/j.biopsycho.2020.107893, PMID: 32437903

[ref33] John P.K. (2000). The Scripture on the Explication of Underlying Meaning. Berkeley, CA: Numata Center for Buddhist Translation and Research.

[ref34] Joyal M. Groleau C. Bouchard C. Wilson M. A. Fecteau S. (2020). Semantic processing in healthy aging and Alzheimer’s disease: a systematic review of the N400 differences. Brain Sci. 10:770. doi: 10.3390/brainsci10110770, PMID: 33114051 PMC7690742

[ref35] Junge C. Boumeester M. Mills D. L. Paul M. Cosper S. H. (2021). Development of the N400 for word learning in the first 2 years of life: a systematic review. Front. Psychol. 12:689534. doi: 10.3389/fpsyg.2021.689534, PMID: 34276518 PMC8277998

[ref36] Kelley N. J. Hortensius R. Schutter D. J. L. G. Harmon-Jones E. (2017). The relationship of approach/avoidance motivation and asymmetric frontal cortical activity: A review of studies manipulating frontal asymmetry. Int. J. Psychophysiol. 119, 19–30. doi: 10.1016/j.ijpsycho.2017.03.001, PMID: 28288803

[ref37] Kim J. J. Cunnington R. Kirby J. N. (2020). The neurophysiological basis of compassion: An fMRI meta-analysis of compassion and its related neural processes. Neurosci. Biobehav. Rev. 108, 112–123. doi: 10.1016/j.neubiorev.2019.10.023, PMID: 31697955

[ref38] Kim J. W. Kim S. E. Kim J. J. Jeong B. Park C. H. Son A. R.. (2009). Compassionate attitude towards others’ suffering activates the mesolimbic neural system. Neuropsychologia 47, 2073–2081. doi: 10.1016/j.neuropsychologia.2009.03.017, PMID: 19428038

[ref39] Koval P. Butler E. A. Hollenstein T. Lanteigne D. Kuppens P. (2015). Emotion regulation and the temporal dynamics of emotions: effects of cognitive reappraisal and expressive suppression on emotional inertia. Cogn. Emot. 29, 831–851. doi: 10.1080/02699931.2014.948388, PMID: 25139315

[ref40] Kutas M. Federmeier K. D. (2011). Thirty years and counting: finding meaning in the N400 component of the event-related brain potential (ERP). Annu. Rev. Psychol. 62, 621–647. doi: 10.1146/annurev.psych.093008.131123, PMID: 20809790 PMC4052444

[ref41] Lopez J. P. P. Pellegrini A. M. (2011). Effective emotional regulation: bridging cognitive science and Buddhist perspective. Enrahonar 47, 139–169. doi: 10.5565/rev/enrahonar/v47.169

[ref42] Lutz A. Brefczynski-Lewis J. Johnstone T. Davidson R. J. (2008). Regulation of the neural circuitry of emotion by compassion meditation: effects of meditative expertise. PLoS One 3:e1897. doi: 10.1371/journal.pone.0001897, PMID: 18365029 PMC2267490

[ref43] Lutz A. Greischar L. L. Perlman D. M. Davidson R. J. (2009). BOLD signal in insula is differentially related to cardiac function during compassion meditation in experts vs. novices. NeuroImage 47, 1038–1046. doi: 10.1016/j.neuroimage.2009.04.081, PMID: 19426817 PMC2854658

[ref44] Lutz J. Herwig U. Opialla S. Hittmeyer A. Jancke L. Rufer M.. (2014). Mindfulness and emotion regulation-an fMRI study. Soc. Cogn. Affect. Neurosci. 9, 776–785. doi: 10.1093/scan/nst043, PMID: 23563850 PMC4040090

[ref45] Mantzios M. Tariq A. Altaf M. Giannou K. (2021). Loving-kindness colouring and loving-kindness meditation: exploring the effectiveness of non-meditative and meditative practices on state mindfulness and anxiety. J. Creat. Ment. Health 17, 305–312. doi: 10.1080/15401383.2021.1884159

[ref46] Meaux E. Roux S. Batty M. (2014). Early visual ERPs are influenced by individual emotional skills. Soc. Cogn. Affect. Neurosci. 9, 1089–1098. doi: 10.1093/scan/nst084, PMID: 23720573 PMC4127009

[ref47] Mitchell J. T. McIntyre E. M. English J. S. Dennis M. F. Beckham J. C. Kollins S. H. (2017). A pilot trial of mindfulness meditation training for ADHD in adulthood: impact on core symptoms, executive functioning, and emotion dysregulation. J. Atten. Disord. 21, 1105–1120. doi: 10.1177/1087054713513328, PMID: 24305060 PMC4045650

[ref48] Morawetz C. Bode S. Baudewig J. Kirilina E. Heekeren H. R. (2016). Changes in effective connectivity between dorsal and ventral prefrontal regions moderate emotion regulation. Cereb. Cortex 26, 1923–1937. doi: 10.1093/cercor/bhv005, PMID: 25631055

[ref49] Murakami H. Katsunuma R. Oba K. Terasawa Y. Motomura Y. Mishima K.. (2015). Neural networks for mindfulness and emotion suppression. PLoS One 10:e0128005. doi: 10.1371/journal.pone.0128005, PMID: 26083379 PMC4471202

[ref50] Newham R. A. (2017). The emotion of compassion and the likelihood of its expression in nursing practice. Nurs. Philos. 18:e12163. doi: 10.1111/nup.12163, PMID: 27982502

[ref51] Nie A. Q. Yu Y. (2021). External (versus internal) facial features contribute most to repetition priming in facial recognition: ERP evidence. Percept. Motor Skills 128, 15–47. doi: 10.1177/0031512520957150, PMID: 32972292

[ref52] Ochsner K. N. Knierim K. Ludlow D. H. Hanelin J. Ramachandran T. Glover G.. (2004). Reflecting upon feelings: an fMRI study of neural systems supporting the attribution of emotion to self and other. J. Cogn. Neurosci. 16, 1746–1772. doi: 10.1162/0898929042947829, PMID: 15701226

[ref53] Onitsuka T. Oribe N. Nakamura I. Kanba S. (2013). Review of neurophysiological findings in patients with schizophrenia. Psychiatry Clin. Neurosci. 67, 461–470. doi: 10.1111/pcn.12090 24102977

[ref54] Reffi A. N. Pinciotti C. M. Darnell B. C. Orcutt H. K. (2019). Trait mindfulness and PTSD symptom clusters: considering the influence emotion dysregulation. Pers. Indiv. Differ. 137, 62–70. doi: 10.1016/j.paid.2018.08.010

[ref55] Riva D. Njiokiktjien C. Bulgheroni S. (2012). Brain Lesion Localization and Developmental Functions: Frontal lobes, Limbic system, Visuocognitive system. London: John Libbey Eurotext Ltd.

[ref56] Rozin P. Royzman E. B. (2001). Negativity bias, negativity dominance, and contagion. Personal. Soc. Psychol. Rev. 5, 296–320. doi: 10.1207/S15327957pspr0504_2

[ref57] Salzberg S. (1995). Lovingkindness: The Revolutionary Art of Happiness. Boston, MA: Shambhala.

[ref58] Salzberg S. (2011). Mindfulness and loving-kindness. Contemp. Buddhism 12, 177–182. doi: 10.1080/14639947.2011.564837

[ref59] Saygin A. P. Sereno M. I. (2008). Retinotopy and attention in human occipital, temporal, parietal, and frontal cortex. Cereb. Cortex 18, 2158–2168. doi: 10.1093/cercor/bhm242, PMID: 18234687

[ref60] Shiah Y. J. (2016). From self to nonself: The nonself theory. Front. Psychol. 7:124. doi: 10.3389/fpsyg.2016.00124, PMID: 26869984 PMC4740732

[ref61] Singh N. Moneghetti K. J. Christle J. W. Hadley D. Plews D. Froelicher V. (2018). Heart rate variability: an old metric with new meaning in the era of using mHealth technologies for health and exercise training guidance. Part one: physiology and methods. Arrhythm. Electrophysiol. Rev. 7, 193–198. doi: 10.15420/aer.2018.27.2, PMID: 30416733 PMC6141929

[ref62] Stenson A. R. Kurinec C. A. Hinson J. M. Whitney P. Van Dongen H. P. A. (2021). Total sleep deprivation reduces top-down regulation of emotion without altering bottom-up affective processing. PLoS One 16:e0256983. doi: 10.1371/journal.pone.0256983, PMID: 34473768 PMC8412406

[ref63] Sullivan R. M. (2004). Hemispheric asymmetry in stress processing in rat prefrontal cortex and the role of mesocortical dopamine. Stress 7, 131–143. doi: 10.1080/102538900410001679310, PMID: 15512858

[ref64] Tang Y. Y. (2018). Brief mindfulness intervention improves emotion regulation in healthy and patient populations. Biol. Psychiatry 83, S58–S59. doi: 10.1016/j.biopsych.2018.02.162

[ref65] Thiruchselvam R. Blechert J. Sheppes G. Rydstrom A. Gross J. J. (2011). The temporal dynamics of emotion regulation: an EEG study of distraction and reappraisal. Biol. Psychol. 87, 84–92. doi: 10.1016/j.biopsycho.2011.02.009, PMID: 21354262

[ref66] Tull M. T. Vidaña A. G. Betts J. E. (2020). “Chapter 10 – Emotion regulation difficulties in PTSD,” in *Emotion in Posttraumatic Stress Disorder*ed. 1st Edn. ed. Tull M.. (San Deigo, CA: Elsevier)

[ref67] van Mol M. M. Kompanje E. J. Benoit D. D. Bakker J. Nijkamp M. D. (2015). The prevalence of compassion fatigue and burnout among healthcare professionals in intensive care units: A systematic review. PLoS One 10:e0136955. doi: 10.1371/journal.pone.0136955, PMID: 26322644 PMC4554995

[ref68] Veerakumar A. Challis C. Gupta P. Da J. Upadhyay A. Beck S. G.. (2014). Antidepressant-like effects of cortical deep brain stimulation coincide with pro-neuroplastic adaptations of serotonin systems. Biol. Psychiatry 76, 203–212. doi: 10.1016/j.biopsych.2013.12.009 24503468 PMC4072754

[ref69] Wu B. W. Y. Gao J. Leung H. K. Sik H. H. (2019). A randomized controlled trial of awareness training program (ATP), a group-based Mahayana Buddhist intervention. Mindfulness 10, 1280–1293. doi: 10.1007/s12671-018-1082-1

[ref70] Zhan J. Wu X. Fan J. Guo J. Zhou J. Ren J.. (2017). Regulating anger under stress via cognitive reappraisal and sadness. Front. Psychol. 8:1372. doi: 10.3389/fpsyg.2017.01372, PMID: 28855881 PMC5557741

[ref71] Zilverstand A. Parvaz M. A. Goldstein R. Z. (2017). Neuroimaging cognitive reappraisal in clinical populations to define neural targets for enhancing emotion regulation. A systematic review. Neuroimage 151, 105–116. doi: 10.1016/j.neuroimage.2016.06.009, PMID: 27288319 PMC5145785

