# Supplementary material for: The neurophysiology of the intervention strategies of Awareness Training Program on emotion regulation
Source: Front Psychol. 2022 Jul 22;13:891656. doi: 10.3389/fpsyg.2022.891656 (PMC9355299; doi:10.3389/fpsyg.2022.891656)

**Table s1.** The heart rate intervals (ms) of the four conditions in the two groups. *NEUT* watching sad pictures with priming of a neutral cue; *WISD* watching sad pictures with priming of a wisdom cue; *COMP* watching sad pictures with priming of a compassion cue; *NORM* watching neutral pictures with priming of a neutral cue; *ATP* Awareness Training Program.

|  | ATP (*M*) | (*SD*) |  | CONTROL (*M*) | (*SD*) |
| --- | --- | --- | --- | --- | --- |
| NEUT | 868.9 | 120.0 |  | 862.7 | 107.0 |
| WISD | 868.9 | 119.5 |  | 860.6 | 108.3 |
| COMP | 866.8 | 119.9 |  | 867.1 | 100.3 |
| NORM | 864.3 | 123.9 |  | 856.7 | 104.3 |

**Table s2.** Demographics of ATP and control groups. No difference was found.

|  | ATP | | CONTROL | | T-test |
| --- | --- | --- | --- | --- | --- |
|  | Mean | SD | Mean | SD | *p* |
| Age | 45.00 | *8.00* | 46.67 | *7.80* | 0.331 |
| Sex | 1.31 | *0.47* | 1.28 | *0.45* | 0.761 |
| Education | 2.88 | *1.09* | 2.84 | *1.19* | 0.860 |
| Marital status | 1.55 | *0.55* | 1.53 | *0.59* | 0.918 |
| Religion | 1.60 | *0.96* | 1.53 | *1.14* | 0.793 |

**Figure s1.** Experiment and analysis pipeline. Please note that the EEG and ECG data was recorded simultaneously but analyzed separately.


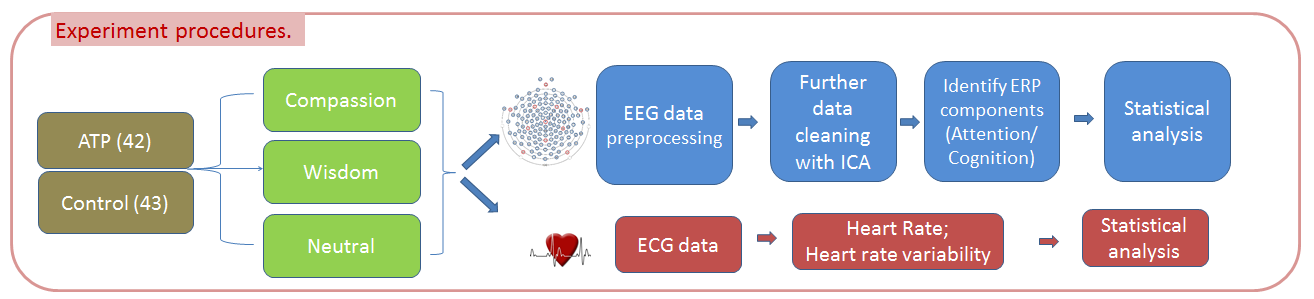


**Figure s2a.** ATP COMP vs ATP NEUT at frontal channel Fz.


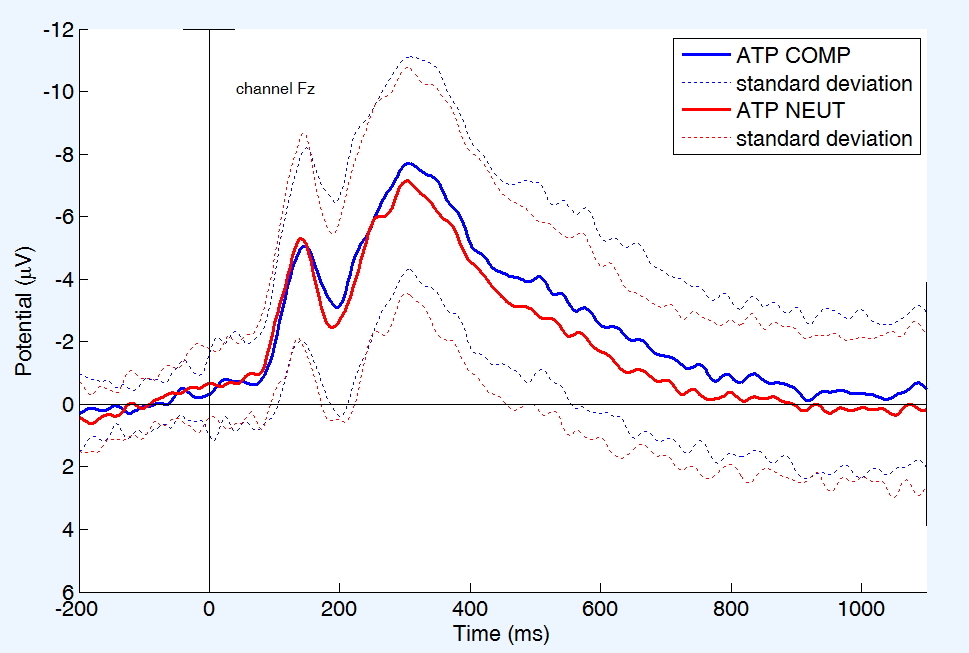


**Figure s2b.** ATP COMP vs ATP NEUT at frontal channel Oz.


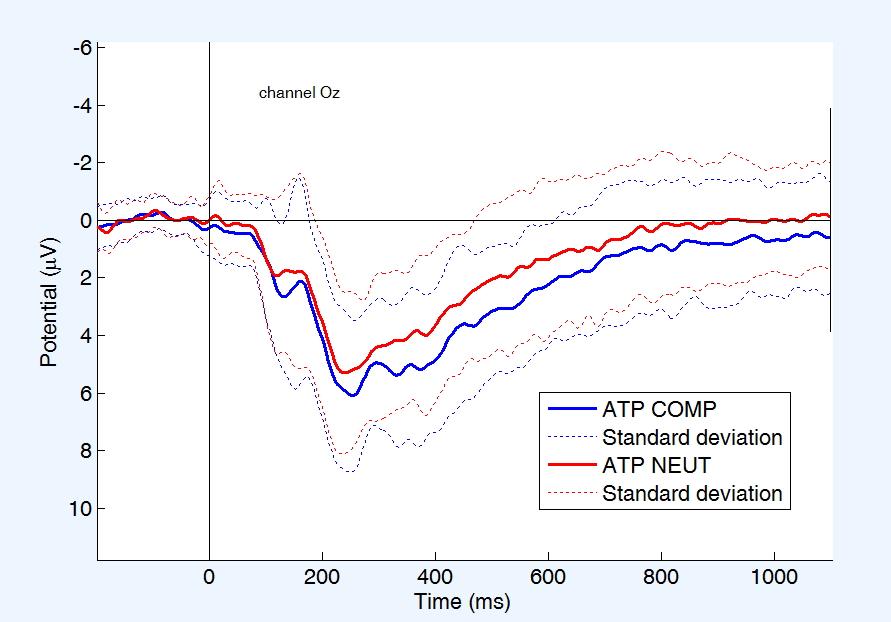


**Figure s3a.** Control COMP vs Control NEUT at frontal channel Fz.


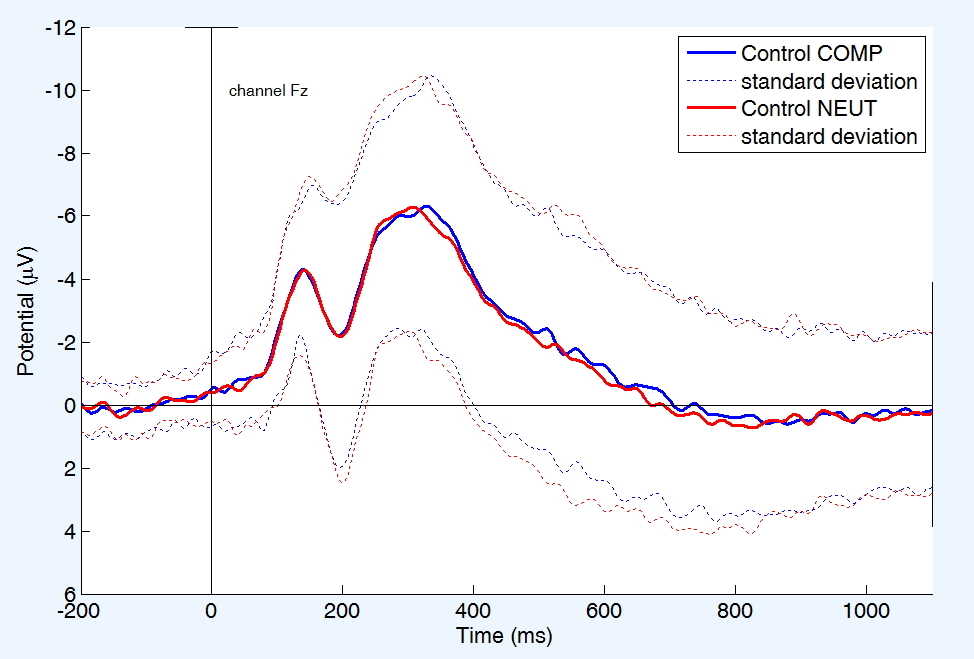


**Figure s3b.** Control COMP vs Control NEUT at frontal channel Oz.


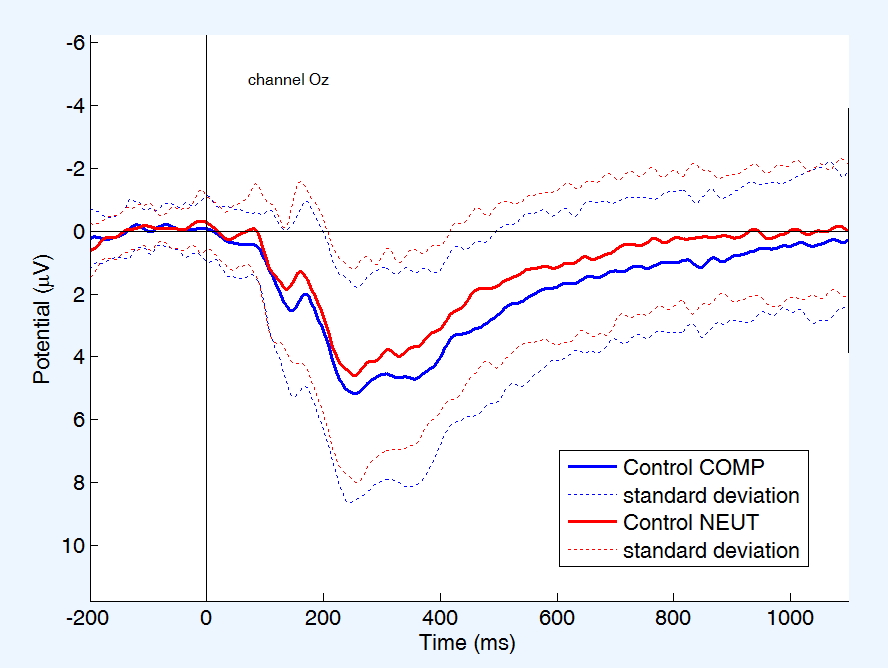


**Figure s4.** The chosen channels for region of interest (ROI) analysis. The left frontal ROI consists of fifteen channels: E12, E13, E18, E19, E20, E22, E23, E24, E26, E27, E28, E29, E33, E34, E35. Result for COMP-NEUT ATP vs. Control at 400-600 ms: Control *M* = -.083 μV, *SD* = 1.599 μV; ATP *M* = -.829 μV, *SD* = 1.741 μV; *t*(83) = 2.057, *p* = .043).


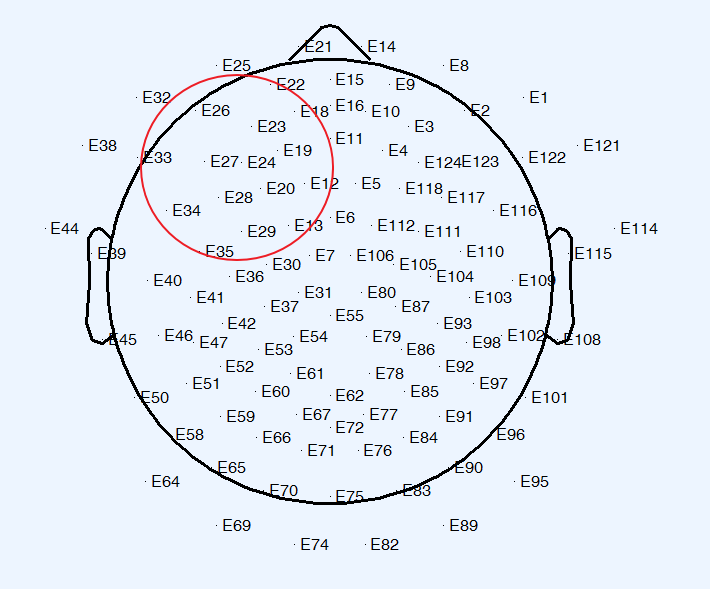


**Figure s5a.** ATP WISD vs ATP NEUT at frontal channel Fz.


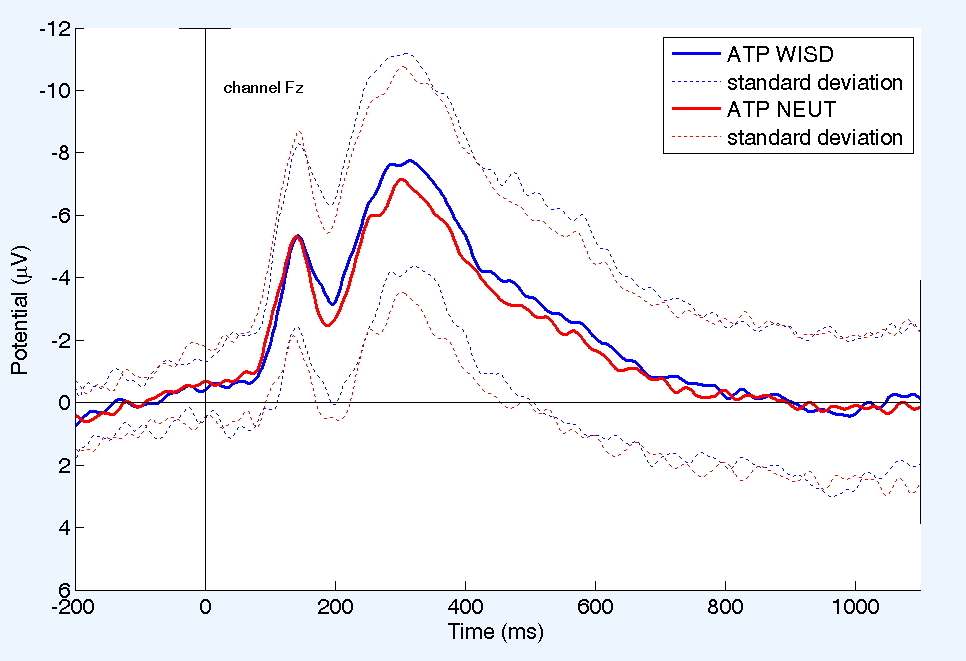


**Figure s5b.** ATP WISD vs ATP NEUT at frontal channel Oz.


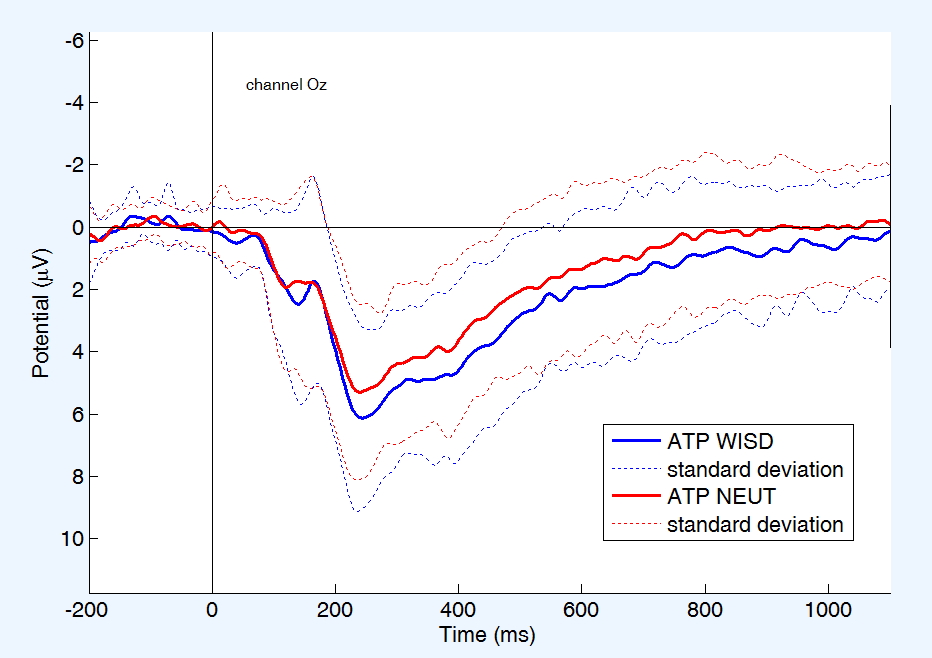


**Figure s6a.** Control WISD vs Control NEUT at frontal channel Fz.


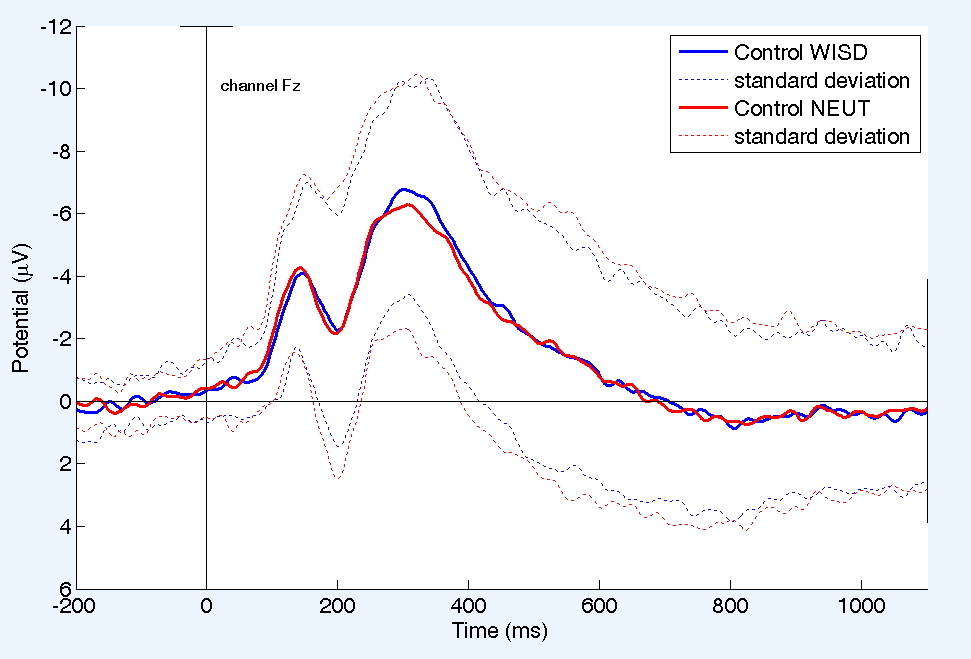


**Figure s6b.** Control WISD vs Control NEUT at frontal channel Oz.


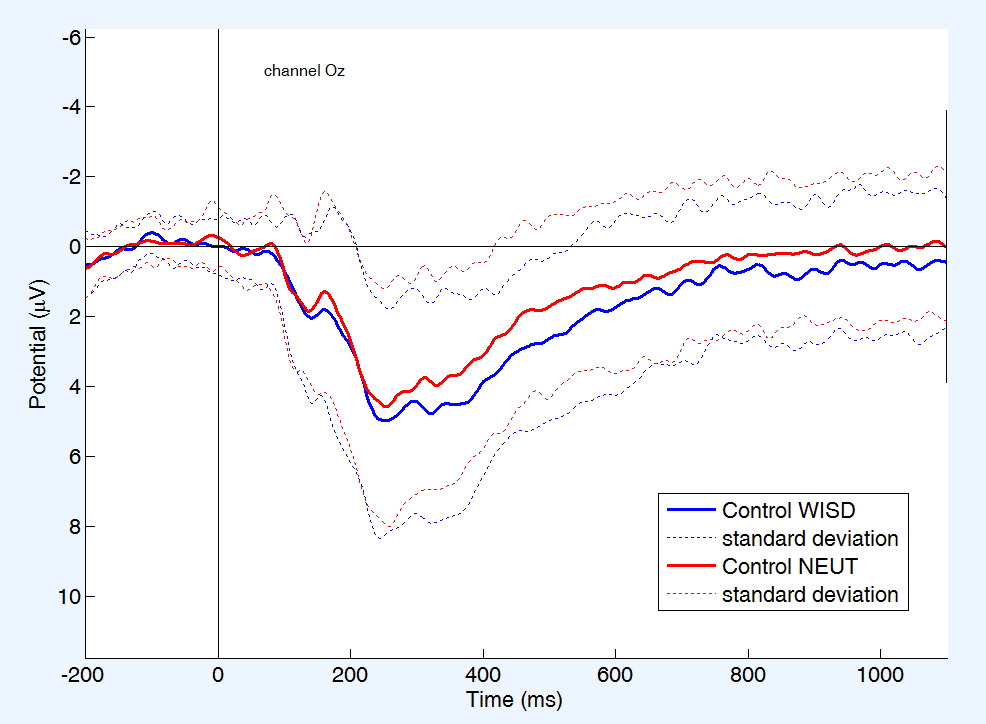


**Figure s7.** The introduction passage written in Traditional Chinese used for priming in the COMP condition. *Translation*: “There are many sadness and helplessness in the world. Please send compassion and good wishes to these people, so that they will be free from suffering and live in peace and happiness!”


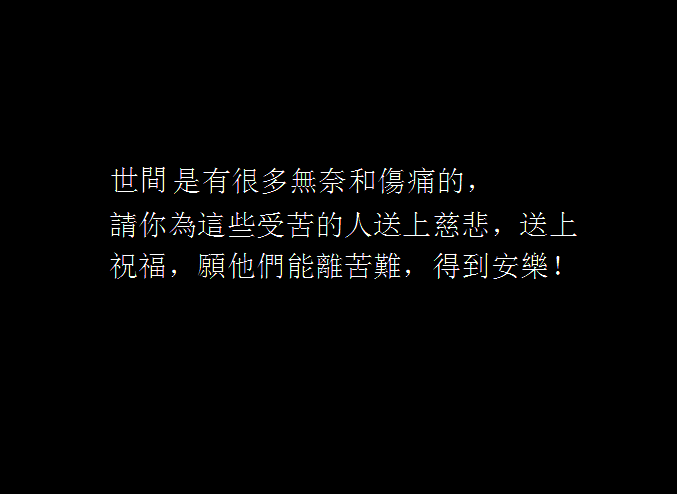


**Figure s8.** The introduction passage written in Traditional Chinese used for priming in the WISD condition. *Translation*: “All worldly phenomena arise and cease as according to causes and conditions, unstable and impermanent. Therefore, the true nature of things that we regard to be happy, suffering, beauty, ugly, joy, sadness and etc. are illusory, mind-only! Hence, we need not be attached to concepts and outer appearances!”


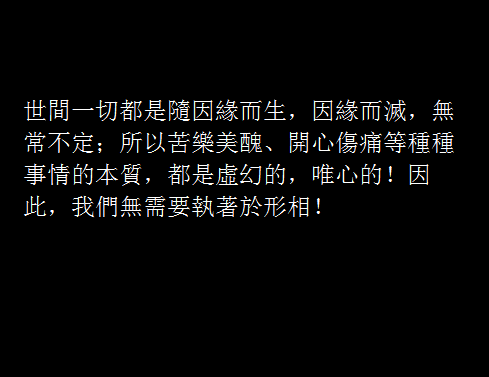


**Figure s9.** The introduction passage written in Traditional Chinese used for priming in the NEUT condition. *Translation*: “You will be seeing images showing ordinary things happened in the real world. Please watch the images carefully.”


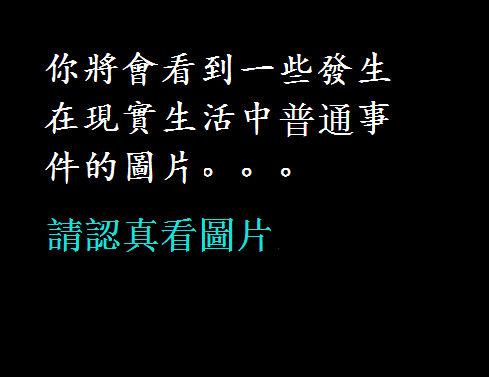

Supplement: Supplementary file 1 [file Data_Sheet_1.docx]
